# Coevolution of Adeno-associated Virus Capsid Antigenicity and Tropism through a Structure-Guided Approach

**DOI:** 10.1128/JVI.00976-20

**Published:** 2020-09-15

**Authors:** L. Patrick Havlik, Katherine E. Simon, J. Kennon Smith, Kelli A. Klinc, Longping V. Tse, Daniel K. Oh, Marco M. Fanous, Rita M. Meganck, Mario Mietzsch, Jürgen Kleinschmidt, Mavis Agbandje-McKenna, Aravind Asokan

**Affiliations:** a Curriculum in Genetics and Molecular Biology, University of North Carolina at Chapel Hill, Chapel Hill, North Carolina, USA; b School of Medicine, University of North Carolina at Chapel Hill, Chapel Hill, North Carolina, USA; c Department of Biochemistry and Molecular Biology, Center for Structural Biology, McKnight Brain Institute, University of Florida, Gainesville, Florida, USA; d Department of Surgery, Duke University School of Medicine, Durham, North Carolina, USA; e Department of Molecular Genetics and Microbiology, Duke University School of Medicine, Durham, North Carolina, USA; f Department of Biomedical Engineering, Duke University, Durham, North Carolina, USA; g Regeneration Next, Duke University, Durham, North Carolina, USA; h German Cancer Research Center, Research Program Infection and Cancer, Heidelberg, Germany; Cornell University

**Keywords:** adeno-associated virus, gene therapy, liver gene delivery, neutralizing antibodies, tropism

## Abstract

Adeno-associated viruses (AAV) are composed of nonenveloped, icosahedral protein shells that can be adapted to package and deliver recombinant therapeutic DNA. Approaches to engineer recombinant capsids for gene therapy applications have focused on rational design or library-based approaches that can address one or two desirable attributes; however, there is an unmet need to comprehensively improve AAV vector properties. Such cannot be achieved by utilizing sequence data alone but requires harnessing the three-dimensional (3D) structural properties of AAV capsids. Here, we solve the structures of a natural AAV isolate complexed with antibodies using cryo-electron microscopy and harness this structural information to engineer AAV capsid libraries through saturation mutagenesis of different antigenic footprints. Each surface loop was evolved by infectious cycling in the presence of a helper adenovirus to yield a new AAV variant that then serves as a template for evolving the next surface loop. This stepwise process yielded a humanized AAV8 capsid (AAVhum.8) displaying nonnatural surface loops that simultaneously display tropism for human hepatocytes, increased gene transfer efficiency, and neutralizing antibody evasion. Specifically, AAVhum.8 can better evade neutralizing antisera from multiple species than AAV8. Further, AAVhum.8 displays robust transduction in a human liver xenograft mouse model with expanded tropism for both murine and human hepatocytes. This work supports the hypothesis that critical properties, such as AAV capsid antibody evasion and tropism, can be coevolved by combining rational design and library-based evolution for clinical gene therapy.

**IMPORTANCE** Clinical gene therapy with recombinant AAV vectors has largely relied on natural capsid isolates. There is an unmet need to comprehensively improve AAV tissue tropism, transduction efficiency, and antibody evasion. Such cannot be achieved by utilizing capsid sequence data alone but requires harnessing the 3D structural properties of AAV capsids. Here, we combine rational design and library-based evolution to coevolve multiple, desirable properties onto AAV by harnessing 3D structural information.

## Abstract (precis)

Clinical gene therapy with recombinant AAV vectors has largely relied on natural capsid isolates. There is an unmet need to comprehensively improve AAV tissue tropism, transduction efficiency, and antibody evasion. Such cannot be achieved by utilizing capsid sequence data alone but requires harnessing the 3D structural properties of AAV capsids. Here, we combine rational design and library-based evolution to coevolve multiple, desirable properties onto AAV by harnessing 3D structural information.

## INTRODUCTION

The process of humanization has proven useful in the development of therapeutic monoclonal antibodies for human use (1). This approach usually represents the modification of nonhuman antibodies to improve their effector functions as well as reduce their immunogenicity. We propose that this paradigm can also be applied to recombinant adeno-associated viral (AAV) vectors, which face similar hurdles that have been highlighted from clinical studies (2, 3). The natural diversity observed within the different AAV clades underscores the potential to further alter the capsid through protein engineering and evolutionary strategies (4, 5). This structural diversity arises primarily from the variable surface loops or regions (VRs) (6). Approaches to engineer AAV have relied on rational domain swapping, DNA shuffling, or peptide insertions (7–9). While these approaches have proven useful for conferring one or two improved properties, for effective humanization of AAV capsids, engineering strategies that comprehensively address distinct functional attributes, such as vector yield/manufacturability, high transduction efficiency, optimal tissue tropism, neutralizing antibody (NAb) evasion, and cross-species compatibility, are warranted.

Using these guiding principles, we adapted an iterative, structure-guided evolution approach to test the hypothesis of whether non-human-derived AAV isolates can be evolved into humanized AAV vectors with improved properties suitable for clinical translation. To achieve such, we relied on structure-function correlates of AAV capsids. Briefly, the AAV capsid is a 25-nm-diameter icosahedral virion with 60 viral protein (VP) subunits (triangulation number, T = 1) (6, 10). The protein shell is composed of three VP subunits (VP1, VP2, and VP3) in the ratio of 1:1:10, each having a conserved jelly roll fold or beta barrel structure with interdigitating loops connecting the beta strands. Single-particle cryo-electron microscopy (cryo-EM) has been utilized to map both receptor and antigenic footprints of different AAV serotypes (10). Receptor usage by different AAV serotypes has been well studied, particularly with regard to cell surface glycans for attachment (11) and, more recently, the essential cellular receptor, KIAA0319L or AAVR (12, 13). In particular, cryo-EM studies have improved our understanding of the mechanisms by which glycans and AAVR are recognized by different AAV serotypes through divergent rules (10, 14–16). The structural models of trimeric AAV capsomers in complex with AAVR show that amino acid residues from different 2/3/5-fold surface loops are critical for this interaction (14–16).

To map antigenic footprints, purified monoclonal antibody fragments (Fabs) and AAV capsids are mixed to create virus-antibody complexes for image data collection and pseudo-atomic resolution models built (17, 18). Each antigenic footprint is comprised of amino acid residues on the capsid surface that are directly in contact with the Fab as well as those occluded by antibody binding. A two-dimensional (2D) roadmap can be used to depict receptor and antigenic footprint residues mapped by cryo-EM and localized to the capsid surface. These studies provide the basis for engineering the AAV capsid surface to potentially achieve improved cross-functionality (18). Here, we harness this structural information to reengineer AAV capsid surface topology and demonstrate that favorable traits, such as human hepatocyte tropism, enhanced gene transfer efficiency, and neutralizing antibody evasion, can be coevolved through infectious cycling.

## RESULTS

### Anti-AAV8 antibodies recognize overlapping capsid antigenic footprints.

**FIG 1:**
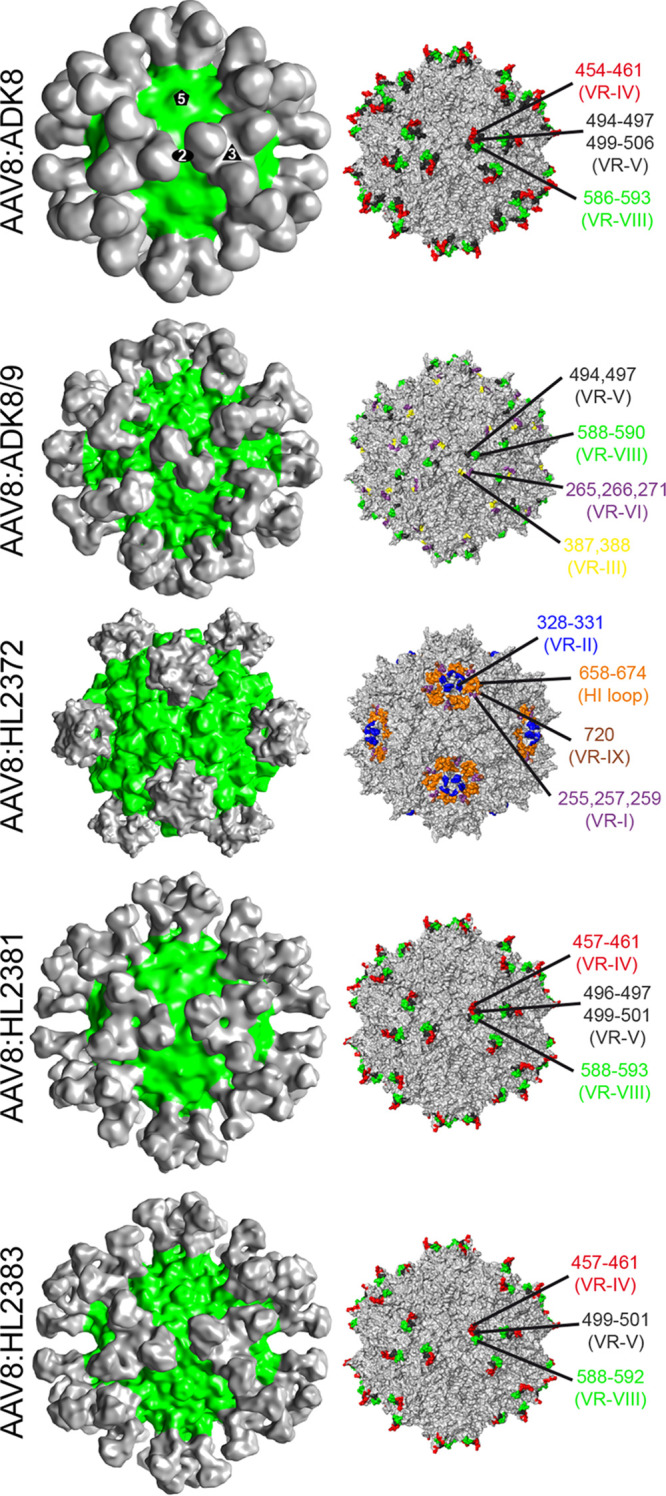
Anti-AAV8 antibody virus complexes. (Left) AAV8-Fab complex reconstructions shown in 3D representation, with the AAV8 capsid colored green and Fabs colored gray. This figure was generated using UCSF-Chimera. (Right) Surface representation of the AAV8 capsid (gray) with the predicted contact residues with Fab highlighted. Capsids are oriented down the 2-fold axis. This image was generated using PyMOL.

To identify the antigenic regions of AAV8 capsids, cryo-electron microscopy (cryo-EM) and image reconstruction were used to determine the three-dimensional (3D) structures of capsid-antibody complexes (18). For this purpose, purified capsids were mixed with purified Fabs at a ratio of 1:120 (capsid to antibody), and the complex was used to collect cryo-EM micrographs for structure determination. Fabs generated from three newly generated mouse monoclonal antibodies (MAbs), HL2372, HL2381, and HL2383 (19), as well as ADK8 and ADK8/9, which were previously described (20), all directed against the AAV8 capsid, were used. These complex structures were determined to ∼12- to 25-Å resolution (Fig. 1). These resolutions were sufficient to generate pseudo-atomic models of the capsid and antibodies into the density maps, enabling the identification of the amino acids forming the antigenic footprint (Table 1). This includes residues predicted to be directly contacted or occluded by the Fab residues. The previously determined AAV8-ADK8 complex structure is shown in Fig. 1A. The AAV8-ADK8/9 complex structure was determined to 17.4-Å resolution (Fig. 1B). The Fab is bound at the side of the 3-fold protrusions and the 2/5-fold wall leaning over the 2-fold icosahedral symmetry axis. Potential contact residues are in VR-I, VR-III, VR-V, and VR-VIII. The AAV8-HL2372 complex structure was determined to 12.5-Å resolution (Fig. 1C). The Fab localizes around the 5-fold axis of symmetry and interacts with the DE loop that includes VR-II, which forms the 5-fold channel, and individual residues in VR-I and VR-IX. Furthermore, the Fab is positioned above the HI loop that forms the floor of the valley surrounding this channel. In the case of HL2381, the Fab bound directly to the tips of the 3-fold protrusions with potential contacts to VR-IV, VR-V, and VR-VIII. The binding characteristics of the Fab are very similar to those described for ADK8 (20). In the HL2383 complex structure (determined to 18-Å resolution; Fig. 1D), the Fab bound almost identically to ADK8 and HL2381 (Fig. 1E) at the 3-fold protrusions, which indicates that this region is highly antigenic for the AAV8 capsid. While the potential contacts for the AAV8-ADK8 complex were VR-IV, VR-V, and VR-VIII, replacement of the amino acid sequences in these surface loops with equivalent amino acids from AAV2 confirmed that residues in VR-VIII (amino acids [aa] 586 to 591) are responsible for ADK8 binding but not VR-IV (aa 456 to 461) and VR-V (aa 493 to 497) (20). Overall, these antibodies bound to common antigenic regions found among the parvoviruses. According to the recently proposed classification system of the parvoviral antibodies, HL2372 would be assigned to group I, ADK8/9 to group II, and ADK8, HL2381, and HL2383 to group III (18).

**TABLE 1:** Summary of cryo-EM reconstructions

AAV:Fab complex	No. of particles used	Resolution (Å)	Correlation coefficient
AAV8:ADK8/9	254	18.5	0.89
AAV8:HL2372	949	12.5	0.83
AAV8:HL2381	53	25.1	0.91
AAV8:HL2383	91	18.0	0.88

### Structural analysis of AAV8 antigenic footprints guides engineering of capsid libraries.

**FIG 2:**
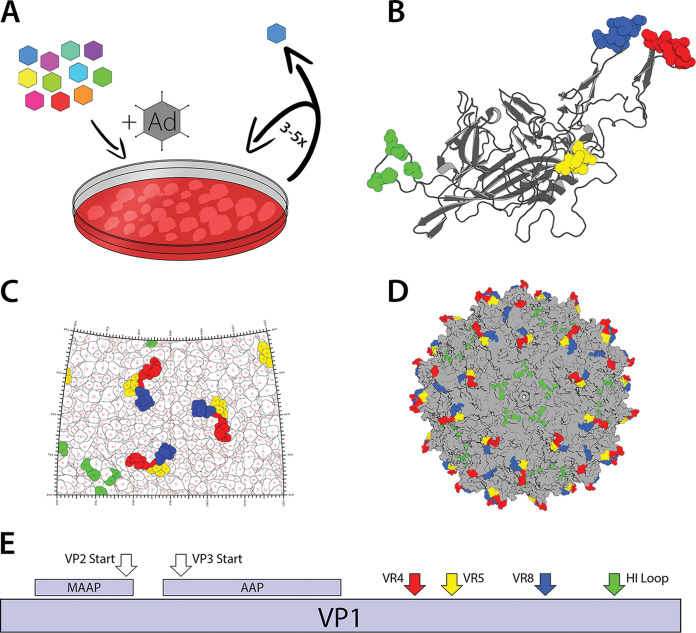
Iterative structure-guided evolution of AAV8. (A) Schematic of iterative mutagenesis and selection. Libraries for each region were created on a lead mutant selected from the previous library and cycled for 3 to 5 rounds on HuH7 cells superinfected with adenovirus. (B to D) Antigenic sites on the AAV8 capsid surface chosen for saturation mutagenesis are colored according to region on an AAV8 monomer and full capsid modeled in PyMol (B and D), as well as a roadmap generated by RIVEM (C). Region IV, region V, region VIII, and the HI loop are highlighted in red, yellow, blue, and green, respectively. (D) Lead mutants from the region V and HI loop libraries were combined to yield the final capsid. (E) Schematic showing proteins encoded by the AAV8 cap gene and the relative locations of the antigenic regions mutated for this study.

The availability of multiple MAbs targeting different regions of the AAV8 capsid used to package and deliver genes in multiple liver-directed gene therapy trials makes it an ideal candidate for generation of capsid libraries. We selected residues on the AAV8 capsid surface implicated as potential contact residues based on the five Fab-capsid complexes described earlier (Fig. 1). Importantly, these clustered antigenic sites are reported to influence multiple functions, including attachment to cell surface glycans as well as AAVR, transduction efficiency, capsid assembly, and genome packaging in different AAV serotypes (18, 21, 22). Thus, we hypothesized that engineering residues implicated in antigenic recognition also influences other biological properties of the AAV capsid. Specifically, we targeted amino acids in variable region IV (red, 455 to 461), variable region V (yellow, 494 to 497), variable region VIII (blue, 586 to 591), and the HI loop (green, 661 to 662, 666 to 667, and 670) highlighted within each monomer VP3 subunit (Fig. 2B). The clustering of these residues on the AAV capsid surface is further illustrated using the 2D roadmap illustration and 3D structural model of the AAV8 capsid as well as the linear schematic of the AAV8 VP1 capsid protein (Fig. 2C to E). We generated saturation mutagenesis libraries within these regions and subjected them to multiple rounds of selection on a human hepatocyte cell line (Fig. 2A). Given the close proximity of these sites to one another in their 3D conformation within the capsid and the potential impact on capsid structural integrity, evolution was carried out in a stepwise fashion to ensure that evolved epitopes would be compatible. The only exception was the HI loop library, which is located farther away at the 5-fold axis of symmetry, which was incorporated independently to yield the final capsid, AAVhum.8.

### Structure-guided evolution yields new AAV8 variants with synthetic VRs.

**FIG 3:**
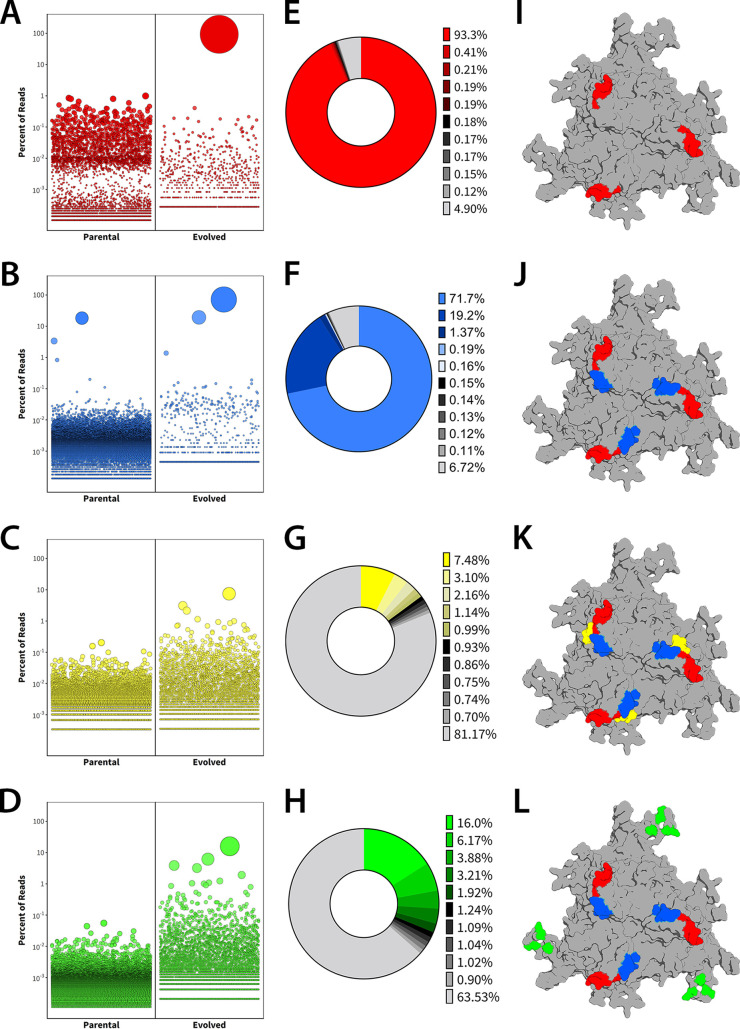
High-throughput sequencing of the parental and evolved libraries was performed with the Illumina MiSeq platform and analyzed with custom Perl scripts. (A to D) Bubble plots showing enrichment of capsid mutants. Each bubble represents a unique amino acid sequence, with bubble size proportional to the percent representation of that sequence in the library. (E to H) Representation of the top 10 mutants from each library as a percentage of total mapped reads. (I to L) AAV8 trimers highlighting regions mutated in iterative evolution strategy.

After selection, parental and evolved libraries for each region were sequenced with the Illumina MiSeq platform. Results were analyzed using a custom Perl script and plotted in R (Fig. 3A to D). The percent representation of most abundant variants for each antigenic epitope (Fig. 3E to H) and the corresponding locations of engineered amino acid residues within the different variable regions is shown on the capsid trimer surface (Fig. 3I to L). Analysis of percent representation of single AAV clones from the different libraries revealed 100- to 1,000-fold enrichment of new variants during each evolution process. Specifically, in the VR-IV library, the variant AAV840 (455-SNGRGV-460) was dominant after three rounds of selection, accounting for ∼93.3% of mapped reads. Interestingly, in addition to the modified sequence, this mutant harbors an amino acid deletion compared to parental AAV8 in this region. Next, we generated the VR-VIII library using the lead AAV840 capsid template and subjected this second library to 3 rounds of evolution. This iterative effort yielded the variant AAV848 (585-GQTQTT-590), which has some sequence similarity to the wild type (586-LQQQNT-591). Importantly, the AAV848 variant, which now carried both VR-IV and VR-VIII modifications, was selected over the AAV840 variant after 3 rounds, accounting for ∼71.7% of mapped reads, compared to ∼19.2% for the latter from this second iterative library.

Next, we generated two independent capsid libraries based on the lead AAV848 variant, one focused on VR-V, which interdigitates between VR-IV and VIII in a neighboring monomer, and the second library based on residues in the HI loop region, which is located around the 5-fold pore. Subjecting these libraries to five rounds of evolution yielded single mutants with reduced dominance compared to VR-IV and VR-VIII lead capsids. In particular, the lead sequence from VR-V accounted for ∼7.5% of mapped reads from that library and ∼15.9% for the HI loop library. To identify leads from these libraries, we developed an algorithm to assess the enrichment of amino acid residues in each position and chose variants with predominant mutations that were selected for in multiple AAV clones (Fig. 3I to L). Thus, the final synthetic capsid variant, AAVhum.8, harbors mutations in VR-IV (455-SNGRGV-460), VR-V (494-YPLQ-497), VR-VIII (585-GQTQTT-590), and the HI loop region (661-RSTFNGDKLN-670).

### Mapping key amino acid preferences within the different synthetic VRs.

**FIG 4:**
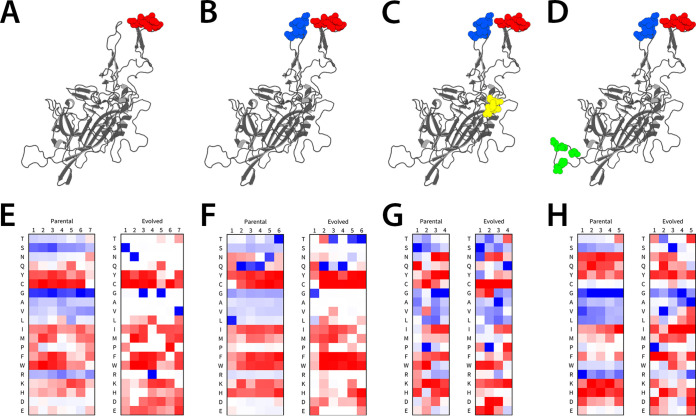
Analysis of amino acid prevalence at each position in the parental and evolved libraries. (A to D) AAV8 monomers showing regions mutated in iterative evolution. (E to H) Heatmap of amino acid frequency at each position of the parental and evolved libraries. The most and least common residues in each position are blue and red, respectively, while the 50th percentile is white. Since the region IV lead mutant was one amino acid shorter here than in the parent, this is represented in panel E as no amino acid being the most common in the 6th position of the evolved library.

A key observation from the aforementioned results is that the different VRs show distinct amino acid preferences at each position that are differentially enriched based on mapped reads. In particular, the VR-V and HI loop libraries did not yield dominant synthetic variants or clones, as with the VR-IV and VR-VIII libraries. To evaluate this trend further, we analyzed the frequency of amino acids at each position by contrasting the representation of amino acids in parental versus evolved libraries. For VR-IV and VR-VIII (Fig. 4A and B), the predominance of a single amino acid sequence after selection is apparent in the heatmaps for the evolved libraries (Fig. 4E and F). The most and least common amino acids at each position are solid blue and red, respectively, while the 50th percentile is white. Since the AAV840 variant is one residue shorter than the parental sequence, the sixth position was selected as not having any preference based on a homology model of the AAV840 variant. Notably, prior to evolution, S, G, and R appear to be the most preferred residues at any position in VR-IV. In contrast, C and hydrophobic residues, such as W, F, and Y, are generally not favored in any position. After multiple rounds of evolution, we observed that S, N, G, R, G, and V were heavily favored in the respective positions, yielding the AAV840 variant (455-SNGRGV-460) (Fig. 4E). Similar trends were observed with VR-VIII (Fig. 4F). The VR-V and HI loop (Fig. 4C and D) libraries generally demonstrated subtler shifts in amino acid preferences (Fig. 4G and H). This implies VR-IV and VR-VIII are more likely to dictate host cell interactions that determine evolutionary pressure. As expected, some biases are seen with the parental libraries, as these sequences were enriched in the earlier evolutionary cycles. One notable example is selection for proline in the second position of the VR-V library, which is maintained during evolution. Other amino acids were strongly represented upon cycling in HuH7 cells, for instance, glutamine in the fourth position of the VR-V library. While the exact functional contributions of these newly evolved amino acid residues in different positions and VRs remain to be determined, these analyses provide a detailed picture of structural determinants on the AAV capsid surface that are driven by functional requirements.

### AAVhum.8 displays enhanced transduction and evades neutralizing antibodies *in vitro*.

**FIG 5:**
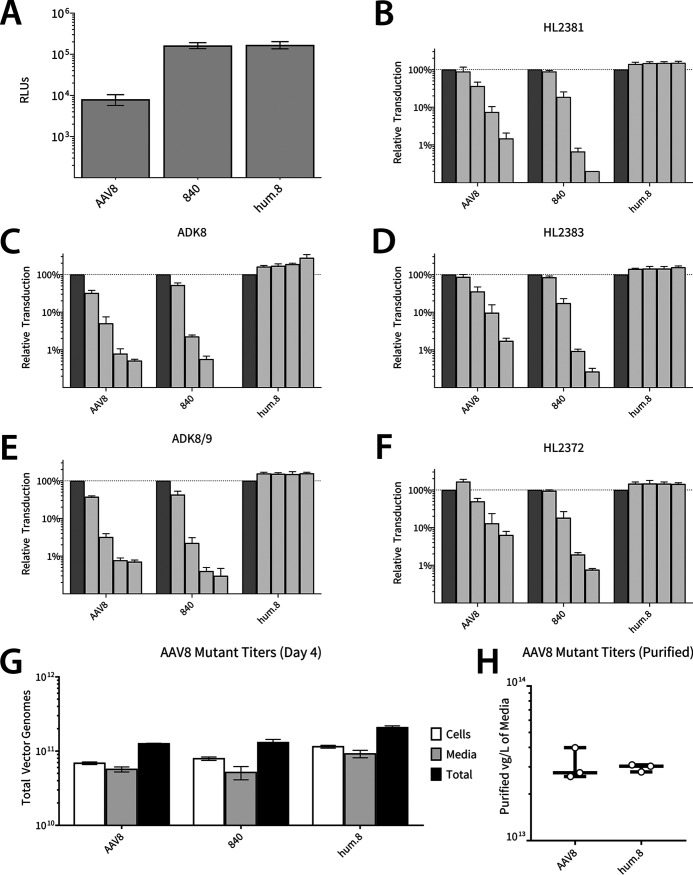
Transduction, antibody escape, and laboratory-scale production properties of AAV8 and evolved variants. (A) Luciferase expression in HuH7 cells transduced with AAV8, 840 (one region modified), or hum.8 (all regions modified) vectors packaging ssCBA-Luc. (B to F) Vectors packaging ssCBA-Luc were incubated with anti-AAV8 neutralizing monoclonal antibodies (2-fold dilutions, 1:3,200 to 1:200) before adding to cells (10,000 vg/cell). Luciferase activity was measured after 48 h and is expressed relative to the no-antibody control (darker bars). (G) AAV8 and mutant vectors were produced by triple plasmid transfection, and vector genome copies in media and cell lysate fractions were determined by quantitative PCR prior to downstream purification. (H) Final purified yields from three laboratory-scale preparations of AAV8 and AAVhum.8 purified directly from cell culture media and subjected to iodixanol gradient ultracentrifugation and desalting (also see Table 2).

The evolved AAV840 mutant derived from the VR-IV library displayed ∼20-fold higher transduction efficiency in the hepatocyte cell line on which it was selected (Fig. 5A). Subsequent modifications upon cycling yielded AAVhum.8, but we did not observe any further change in transduction efficiency. We then assessed the ability of AAVhum.8 to be neutralized by different anti-AAV8 monoclonal antibodies (MAbs) derived in mice. As described earlier (Fig. 1), three newly generated mouse MAbs, HL2372, HL2381, and HL2383 (19), as well as ADK8 and ADK8/9, which have been previously described (20), all directed against the AAV8 capsid, were used. Specifically, as described for Fig. 1, ADK8 has been shown to bind and neutralize AAV8 capsids in the VR-IV, VR-V, and VR-VIII regions, while ADK8/9 cross-reacts with both AAV8 and AAV9 capsids. HL2381 and HL2383 recognize these 3-fold symmetry spike regions as well, while HL2372 binds residues at the 5-fold symmetry axes, including the HI loop region. Evolution of the VR-IV antigenic epitope alone, while able to confer increased potency, was insufficient for enabling escape from anti-AAV8 MAbs in general (Fig. 5B to F). In contrast, AAVhum.8, which carries composite mutations in distinct antigenic regions (VR-IV, V, VIII, and the HI loop), efficiently evades anti-AAV8 MAbs at different dilutions while affording high transduction levels *in vitro*. Furthermore, mutant capsids at each stage of evolution displayed relative vector genome titers similar to those of AAV8 in cell lysate and culture media (Fig. 5G). The total yields of purified vector in three laboratory-scale preparations of AAV8 and AAVhum.8 in adherent 293 cells were comparable (Fig. 5H and Table 2). Importantly, vectors for these preparations were purified by only harvesting cell culture media, supporting the notion that AAVhum.8 is secreted into the media like parental AAV8 vectors.

**TABLE 2:** Titers of AAV8 and AAVhum.8 vectors produced at laboratory scale

Virus and transgene	Harvested media (ml)	Total yield (vg)	Comparative yield (vg/liter) media
AAV8			
ssCBA-Luc	200	5.2E + 12	2.60E + 13
sc-CBh-GFP	160	6.39E + 12	3.99E + 13
sc-CBh-GFP	400	1.1E + 13	2.75E + 13
Hum.8			
ssCBA-Luc	200	5.56E + 12	2.78E + 13
sc-CBh-GFP	160	4.95E + 12	3.09E + 13
sc-CBh-GFP	400	1.21E + 13	3.03E + 13

**FIG 6:**
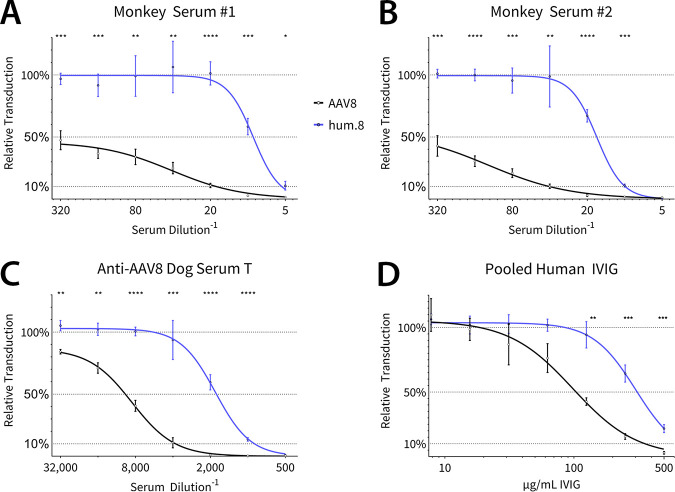
Neutralization and escape profiles of AAV8 and AAVhum.8 with polyclonal antibodies and sera. Vectors were preincubated with individual monkey sera (A and B), serum from a beagle treated with AAV8 vector (C), or pooled human intravenous immunoglobulin (D) before adding to HuH7 cells (100,000 vg/cell). Luciferase expression was measured after 24 h.

In addition to murine MAbs, we evaluated anti-AAV8 polyclonal antisera from rhesus macaques and a beagle previously injected with AAV8 vectors during preclinical evaluation (Fig. 6A to C). Evaluation of AAVhum.8 and AAV8 vectors packaging a CBA promoter-driven luciferase reporter transgene revealed a 2- to 6-fold improvement against this polyclonal serum panel. In addition, both vectors were tested against pooled human IgG, where a similar shift in the neutralization was observed (Fig. 6D). These results demonstrate that antigenic footprints determined by structural studies with mouse-derived MAbs overlap those recognized by polyclonal sera from large animal models as well as humans. Importantly, AAVhum.8 is able to evade NAbs across multiple species, demonstrating potential for preclinical-to-clinical translation.

### AAVhum.8 demonstrates improved transduction profile in a chimeric humanized liver model *in vivo*.

**FIG 7:**
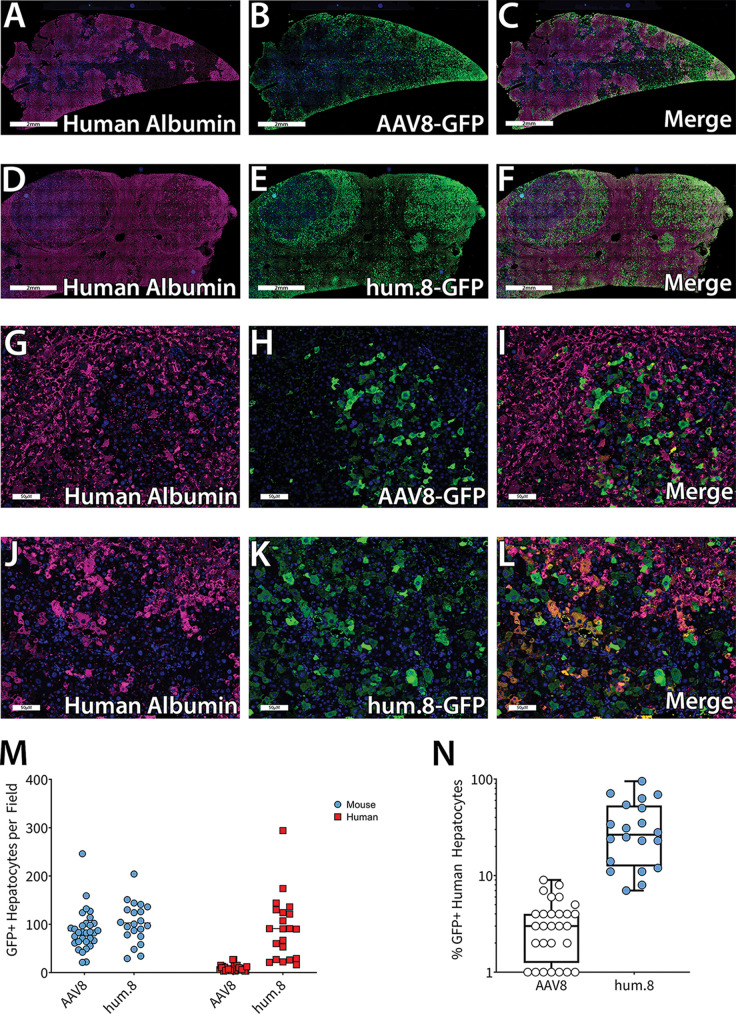
Evaluation of AAVhum.8 in a humanized mouse model. FAH^−/−^ humanized liver mice were injected with AAV8 or AAVhum.8 vector expressing scCBh-GFP at a dose of 1e12 vg. Livers were collected 3 weeks postinjection and immunostained for human albumin and GFP. Confocal fluorescence images are shown at 2 mm (A to F) and 50 μm (G to L); magnification, ×40. For each animal, 10 fields of view were examined to count the number of GFP-positive murine and human hepatocytes (M) and the percentage of total human hepatocytes that were GFP^+^ (N). Each data point represents one field of view. *n* = 3 for AAV8, *n* = 2 for AAVhum.8.

Next, to test if the increased human hepatocyte transduction observed *in vitro* is recapitulated *in vivo*, we evaluated AAV8 and AAVhum.8 vectors in Fah^−/−^/Rag2^−/−^/Il2g^−/−^ (FRG) humanized liver chimeric mice (Yecuris). Vectors packaging scCBh-eGFP were delivered intravenously via retro-orbital injection at a dose of 1 × 10^12^ vector genomes (vg)/mouse (5 × 10^13^ vg/kg of body weight). Livers were collected 3 weeks postinjection and immunostained for the presence of human or mouse albumin as well as green fluorescent protein-positive (GFP^+^) cells. Images obtained using confocal fluorescence microscopy revealed that similar to results published by other groups, AAV8 preferentially transduced zones rich in murine hepatocytes (Fig. 7A to C and G to I [×40 magnification]), while AAVhum.8 transduced human and mouse hepatocyte zones equally well (Fig. 7D to F and J to L [×40 magnification]). The number of GFP^+^ mouse and human hepatocytes (Fig. 7M) as well as the percentage of total GFP^+^ human hepatocytes are shown (Fig. 7N). AAVhum.8 transduced numbers of murine hepatocytes similar to those of parental AAV8; however, it is important to note that AAVhum.8 showed a 10-fold increase in the total number of transduced human hepatocytes. In terms of percentage of total human hepatocytes transduced, AAV8 transduced an average of ∼3.39%, while AAVhum.8 transduced ∼34.4%.

## DISCUSSION

Structural studies of viruses have revolutionized our understanding of how AAV capsids interact with host receptors and antibody-mediated neutralization mechanisms (10, 18). The current study hinges on cryo-EM reconstruction of AAV capsids complexed with MAbs that sheds light on both antigenic and tropism determinants. We leveraged this information to engineer AAV capsid libraries and evolve new variants with the goal of humanizing natural serotypes for gene therapy applications. Successful clinical application requires recombinant AAV vectors that can not only transduce better but also demonstrate selective tissue tropism, NAb evasion, and cross-species compatibility for translational studies. Most engineering strategies described to date rely on peptide insertion, generation of mosaic capsids by DNA shuffling, or *in silico* alteration of different AAV clades based on phylogenetic analysis (8, 9). These approaches primarily rely on capsid protein or DNA sequence data, which is either restricted to manipulation of a single surface domain or domain/residue swapping between naturally occurring AAV strains (ancestral or extant). While capable of improving one or two attributes, it is difficult to comprehensively address antigenicity, tropism, and transduction efficiency with such approaches. Here, we demonstrate the ability to simultaneously improve upon these limitations through a stepwise, structure-guided approach that enables coevolution of different functions. By analyzing cryoreconstructed images of AAV8 capsid-antibody complexes, we first determined clusters of amino acid residues for saturation mutagenesis to generate capsid libraries. Mutating these key surface loop residues disrupted antigenic footprints on the AAV8 capsid. Synergistically, when rescued through coinfection with adenovirus, these mutations also conferred improved transduction efficiency and liver tropism.

At the structural level, our results underscore the plasticity of the AAV capsid surface. When using a precisely directed, structure-guided approach, it is evident that novel amino acid sequences that do not exist in natural AAV isolates can be evolved, as observed with each newly evolved antigenic epitope on AAVhum.8. In attempting to take the first steps toward establishing structure-activity relationships, we mapped the relative enrichment of specific amino acids in each position within the surface loops. As mentioned earlier, the diversity of amino acids that can be tolerated within AAV surface loops is evident. While it remains challenging to determine the potential role of each amino acid within each evolved footprint, it is possible to assess the extent of selective pressure(s) exerted on each amino acid position. These evolutionary parameters could include proper folding of VPs, capsid assembly, which relies on interdigitation of surface loops, genome packaging, cell surface binding and entry, and/or postentry trafficking (18, 21). Notably, our high-throughput analysis data imply that selective pressure diminishes after optimal residues for VR-IV and -VIII are first evolved through repeated infectious cycling of AAV libraries with adenoviral coinfection. Consequently, VR-V and the HI loop, which were evolved in a mutually exclusive manner, appear to tolerate a broader spectrum of mutations. Although we did not observe improvements in transduction upon evolving the latter two antigenic epitopes, they unequivocally contributed to NAb evasion. Thus, while all the evolved footprints contribute to antigenicity, it is evident from our results that selective pressure exerted on the 3-fold symmetry axis is more likely to yield coevolved traits, such as transduction efficiency and tropism.

Our results also validate the hypothesis that capsid antigenicity and functional properties of AAV capsids, such as tropism and transduction, overlap in the structural context. The synthetic variant AAVhum.8 not only confirms this notion but also highlights the robustness of our structure-guided evolution approach. Specifically, AAVhum.8 has four antigenically distinct regions that were selected by stepwise evolution on human hepatocyte cultures. We validated that these novel surface footprints were less recognized by NAbs from different species than otherwise neutralized parental AAV8 capsids. In particular, we demonstrated that AAVhum.8 was less neutralized by anti-AAV8 polyclonal serum from primates and dogs treated previously with different AAV8 vectors. Moreover, AAVhum.8 displayed a distinct, improved immune escape when treated with pooled human IgG compared to AAV8. Concomitantly, AAVhum.8 also displays a markedly improved transduction profile compared to those of AAV8 vectors in human hepatocyte cultures *in vitro* and humanized chimeric mouse livers as well. In particular, we first observed that AAV8 preferentially transduces murine over human hepatocytes, consistent with earlier reports. On the other hand, AAVhum.8 shows expanded tropism for human hepatocytes while continuing to transduce mouse cells. These observations underscore that antigenic and tropism determinants overlap on the AAV capsid surface and can indeed be coevolved while imparting improved functionality.

Further, while it was not necessarily expected that an AAV capsid evolved to transduce human hepatocytes would retain murine transduction capability, the synthetic variant AAVhum.8 presents the opportunity to streamline bench-to-clinic translation through the use of a single AAV vector from mouse models to patients. In summary, the aforementioned observations corroborate the notion that the antigenically distinct footprints on the AAVhum.8 capsid have coevolved to efficiently transduce hepatocytes upon repeated experimental adaptation. Consequently, AAVhum.8 vectors show promise for liver-directed gene therapy and gene editing while evading neutralizing antibodies. It is noteworthy that this structure-driven approach potentially can be utilized to improve upon the antibody-evading potential of other natural liver-tropic AAV (23, 24) or humanized liver-tropic AAV variants engineered by other groups (25, 26). Further, although appropriate models would be required, our approach potentially can be extended to humanize AAV to effectively target and transduce other human organs, such as the heart, muscle, pancreas, kidney, and central nervous system, while simultaneously diminishing immune recognition. We also postulate the potential to coevolve cross-species compatibility, which is a potentially useful trait that enables reliable translation from mouse models of disease to humans.

## MATERIALS AND METHODS

### AAV8 VLP production and purification.

AAV8 virus-like particles (VLPs) were expressed using a recombinant baculovirus expressing the AAV8 capsid viral proteins VP1, VP2, and VP3, as previously reported (19). Briefly, the baculovirus was used to infect insect *Sf*9 suspension cells and incubated for 72 h at 27°C. Cells were harvested and pelleted by centrifugation at 2,000 × *g* for 15 min at 4°C. Pellets were resuspended in 1× TD buffer (137 mM NaCl, 10 mM Na_2_HPO_4_, 1.7 mM KH_2_PO_4_, 5.3 mM KCl, 1 mM MgCl_2_ at pH 7.4) and subjected to three freeze/thaw cycles to release the VLPs. Benzonase (New England BioLabs) was added, and the crude lysate was incubated at 37°C for 1 h. VLPs released into the culture medium were recovered by precipitation with 10% (wt/vol) polyethylene glycol (PEG) 8000 and pelleted at 9,000 × *g* for 90 min at 4°C, and the pellet was resuspended and combined with the crude lysate supernatant. The sample was centrifuged at 10,000 × *g* for 20 min at 4°C to remove cellular debris. The supernatant was collected and diluted with TNET (50 mM Tris, 100 m NaCl, 1 mM EDTA, and 0.2% Triton X-100 at pH 8) and pelleted through a 20% sucrose (in TNET) cushion via ultracentrifugation at 149,000 × *g* for 3 h at 4°C. The resulting pellet was resuspended at 4°C in TNTM (25 mM Tris, 100 mM NaCl, 0.2% Triton X-100, 2 mM MgCl_2_ at pH 8), loaded onto a sucrose-step gradient (5% to 40%, wt/vol), and spun at 151,000 × *g* for 3 h at 4°C. After centrifugation, a band visible under bright light was extracted from the 20/25% sucrose layer and dialyzed into 20 mM Tris-HCl–250 mM NaCl at pH 7.5. The band fraction was concentrated using a 150-kDa molecular weight cutoff (MWCO) Apollo column. Purified VLPs were examined by SDS-PAGE to check for the presence of VP1, VP2, and VP3, and the approximate VLP concentrations, in milligrams per milliliter, were determined using optical density readings at 280 nm with an extinction coefficient of 1.7. VLP integrity was confirmed by applying 5 μl of purified sample onto carbon-coated EM grids (EMS), staining with 2% uranyl acetate, and viewing using an FEI (now ThermoFisher) Spirit 120 kV transmission electron microscope (TEM).

### Fab generation and purification.

The production of the AAV8 MAbs from which Fabs were generated for these studies has been reported (19). The production of Fabs was as previously described (22). Briefly, the four AAV8 MAbs were purified from hybridoma supernatants by affinity chromatography using protein G-Sepharose columns (GE Healthcare). Antibodies eluted were concentrated using a 20-kDa MWCO Apollo 7-ml centrifugal concentrator (Orbital Biosciences). Immobilized papain was activated with l-cysteine according to the manufacturer’s instructions (Pierce, Rockford, IL), mixed with purified MAbs at a suggested enzyme/substrate ratio of 1:160 (wt/wt), and incubated with moderate shaking at 37°C overnight. The reaction was stopped with 10 mM Tris-HCl, pH 7.5, and then gently centrifuged (200 × *g*, 5 min, 4°C) to pellet the immobilized papain-agarose beads. The aqueous mixture was carefully removed and diluted with 20 mM sodium phosphate buffer (pH 8.5) and applied to a Hi-Trap protein A column (GE Healthcare). Fabs were collected in the flowthrough and concentrated using a 9-kDa MWCO Apollo 7-ml centrifugal concentrator (Orbital Biosciences). The purity of the samples was monitored by SDS-PAGE.

### Cryo-EM data collection on the VLP-Fab complexes.

Purified VLPs were mixed with Fabs at a ratio of 1:120 (capsid to antibody). Complexes were viewed on an FEI Spirit 120-kV TEM by negative-stain electron microscopy to confirm Fab decoration of the VLPs prior to sample vitrification. Three microliters of the AAV8-Fab complex samples was applied to glow-discharged C-Flat (CF-2/2 to 4c-50; Protochips, Inc.) holey carbon grids, blotted, and vitrified in liquid ethane using a Vitrobot Mark 4 (FEI) at 95% humidity and 4°C. The vitrified grids were stored in liquid nitrogen until data collection. For cryo-EM data collection, grids were transferred into Gatan cryosample holders and placed into an FEI Tecnai TF20 cryoelectron microscope operating at 200 kV. The complex data were collected at a magnification of 67,050× with a 1.5- to 3.0-μm defocus range under low-dose conditions (∼20 electrons/Å^2^) using a Gatan UltraScan 4000 charge-coupled device camera at 1.81 Å/pixel.

### 3D reconstruction of the AAV8-Fab complexes.

Individual AAV8-Fab particle images were manually extracted from each micrograph using the EM software package (27) (http://cryoEM.ucsd.edu/programs.shtm). Preprocessing of the selected images (normalization and apodization) and estimation of the defocus level of each micrograph were performed using the AUTOPP subroutine (options F, O, and 3×) of AUTO3DEM, as previously described (27). The *ab initio* random-model computation procedure in AUTO3DEM was used to generate a starting model of the virus-Fab complexes at an ∼30-Å resolution from 10% of the total boxed particle images. This map was used to initiate full orientation and origin determination and refinement of the entire set of images with AUTO3DEM utilizing the gold standard protocol. Corrections to compensate for the effects of phase reversals in the microscope contrast-transfer function for each micrograph were performed as recently described, without amplitude corrections (28). The resolutions of the final reconstructions were calculated on the basis of a Fourier shell correlation (FSC, 0.5).

### Docking of the AAV8-Fab model in the cryo-EM maps and epitope prediction.

The atomic coordinates of the AAV8 capsid (60mer generated from PDB accession number 2QA0 [29]) using the VIPERdb online server (30) were docked into the cryoreconstructed density maps using the “fit in map” subroutine in UCSF-Chimera (31) and used to subsequently determine the absolute scale (i.e., pixel size) of the cryo-EM map by correlation coefficient (CC) calculations. The pixel size of the reconstructed map was adjusted using the e2proc3d.py subroutine in EMAN2 (32), and a ccp4 format map was generated with the Mapman program (33). A generic Fab structure (PDB entry 2FBJ) was fitted into the density on the surface of the capsid and a fully decorated AAV8-Fab model generated using VIPERdb. The “fit-in-map” function in Chimera was used to obtain the CC of the fitted complex models relative to the maps. The interfacing amino acids between the AAV8 capsid and the docked Fab coordinates, for each complex, were analyzed using the PDBe Protein Interfaces, Surfaces, and Assemblies (PISA) service at the European Bioinformatics Institute (http://www.ebi.ac.uk/pdbe/prot_int/pistart.html) to predict the defined epitopes in the form of interacting contact residues.

### Cells, viruses, and antibodies.

HEK293 and HuH7 cells were cultured in Dulbecco’s modified Eagle’s medium (DMEM) supplemented with 10% or 5% fetal bovine serum (FBS), respectively, 100 U/ml penicillin, and 10 μg/ml streptomycin in 5% CO_2_ at 37°C. Human adenovirus 5 (Ad5) was purchased from the American Type Culture Collection. Mouse anti-AAV8 MAbs ADK8, ADK8/9, HL2381, HL2383, and HL2372 have been described previously (19, 20). Naïve human serum samples were purchased from Valley Biomedical. Rhesus macaque and dog serum samples from animals pretreated with AAV8 vectors from unrelated studies were gifts from the Oregon Health & Science University and University of Washington. All serum samples were heat inactivated at 55°C for 15 min before use.

### AAV vector production, purification, and quantification.

Recombinant AAV vectors were produced by transfecting HEK293 cells at 70 to 80% confluence with polyethylenimine using the triple-plasmid transfection protocol (34). Recombinant vectors packaging single-stranded genomes encoding firefly luciferase driven by the chicken β-actin promoter (ssCBA-Luc) or self-complementary green fluorescence protein driven by a hybrid chicken β-actin promoter (scCBh-GFP) were generated using this method. Subsequent steps involving the harvesting of recombinant AAV vectors and downstream purification were carried out as described earlier (35). Recombinant AAV vector titers were determined by quantitative PCR with primers amplifying AAV2 inverted terminal repeat regions (ITRs) 5′-AACATGCTACGCAGAGAGGGAGTGG-3′ and 5′-CATGAGACAAGGAACCCCTAGTGATGGAG-3′.

### Generation of AAV capsid libraries.

AAV capsid libraries were engineered through saturation mutagenesis of residues within antigenic epitopes identified through cryo-EM. The AAV8 region IV library was constructed as previously described (36). Briefly, a library insert spanning the BsiWI and SbfI sites in the AAV8 Cap, containing degenerate nucleotides at the selected region, was ligated into pTR-AAV8**. The pTR-AAV8** plasmid contains AAV2 Rep and AAV8 Cap flanked by AAV2 ITRs, with amino acids 446 and 447 in the AAV8 Cap mutated to stop codons to reduce wild-type AAV8 plasmid contamination. Subsequent libraries differed only in the generation of the library inserts and vectors. The region V, region VIII, and HI loop library inserts were constructed through overlap extension of two PCR products. The first product spans the first restriction site and ends immediately before the library region. The second product contains 30 bp of overlap with the first, followed by the degenerate nucleotides of the library, and spans the second restriction site. PCR and overlap extension reactions were performed using Q5 polymerase (New England Biolabs). Of note, the PCR products used in the overlap extension reactions were amplified using modified AAV8 plasmids, created through site-directed mutagenesis, as the template. For each library, a null AAV8 template plasmid was created by replacing the selected region with a sequence of the same length containing stop codons in every reading frame. Additionally, two cap-null AAV2/8 ITR plasmids were constructed by replacing the region corresponding to each respective library insert with GFP flanked by the appropriate restriction sites. Thus, the AAV8 template plasmid used to generate each library insert did not contain a wild-type sequence at the library site, and the AAV8 ITR plasmid ligated with the library insert contained no portion of the insert.

### Evolution of humanized AAV8 mutants.

To generate the viral libraries, HEK293 cells were transfected as described above, except that equal molar ratios of pTR-AAV8-Library and adenovirus helper plasmid pXX680 were used rather than the triple plasmid protocol used to produce AAV vectors. HuH7 cells were cultured to ∼75% confluence and infected overnight with AAV8 libraries at 5,000 viral genomes per cell. The following day, the culture medium was replaced with medium containing Ad5 at a multiplicity of infection of 0.5. At 50% to 75% cytopathic effect, the supernatant was collected and incubated at 55°C for 30 min to inactivate the Ad5. DNase I-resistant viral genomes in the media were quantified and served as the inoculum for each subsequent round of infection. For each library, 3 to 5 cycles were completed before isolation of single clones and high-throughput sequencing of capsid mutants.

### Identification of newly evolved AAV8 mutants.

To analyze the sequence diversity of the parental and evolved AAV CAM libraries, DNase I-resistant viral genomes were isolated from media and amplified by Q5 polymerase for 10 to 18 cycles (New England BioLabs) using primers 5′-CCCTACACGACGCTCTTCCGATCTNNNNNGTACCTGTACTACTTGTCTCG-3′ and 5′-GACTGGAGTTCAGACGTGTGCTCTTCCGATCTNNNNNAGACCATACCGGGTAAG-3′ for regions IV and VIII, 5′-CCCTACACGACGCTCTTCCGATCTNNNNNGAATCCTCTGATTGACCAGTACCTGTACTACTTGTCTCGG-3′ and 5′-GACTGGAGTTCAGACGTGTGCTCTTCCGATCTNNNNNGGTTCTGCCAGACCATACCGGGTAAGG-3 for region V, and 5′-CCCTACACGACGCTCTTCCGATCTNNNNNCATCCTCCGCCTCAGATCCTGATCAAGAACACG-3′ and 5′-GACTGGAGTTCAGACGTGTGCTCTTCCGATCTNNNNNCAGATTACGGGTGAGGTAACGGGTGCC-3′ for the HI loop. The Illumina MiSeq sequencing adaptor for multiplexing was added in a second round of PCR using Q5 polymerase with P5 and P7 primers. After each round of PCR, the products were purified using the PureLink PCR Micro kit (Invitrogen). The quality of the amplicons was verified using a Bioanalyzer (Agilent), and concentrations were quantified using a Qubit spectrometer (Invitrogen). Libraries were then prepared for sequencing with the Illumina MiSeq 300 reagent kit v2 by following the manufacturer’s instructions and sequenced on the MiSeq system.

### Sequencing data analysis.

Demultiplexed reads were analyzed with an updated in-house Perl script (36). In brief, reads were probed for the nucleotide sequences corresponding to each library region, and the occurrence of each nucleotide sequence was counted and ranked. These sequences were also translated, and the resulting amino acid sequences were counted and their percent representation calculated and ranked. Sequences were plotted according to their representation in each library using the R graphics package v3.5.2. A second Perl script was used to calculate the amino acid representation at each position in each library, taking into account the contribution of each mutant in the library. These data were plotted in heat map form in GraphPad Prism.

### Isolation of individual AAV8 mutants.

Single clones from each library were isolated for further characterization. DNase I-resistant genomes were amplified from media after evolution by Q5 polymerase (New England BioLabs) using primers flanking the BsiWI and SbfI sites (regions IV, V, and VIII) or SbfI and an XbaI site between the cap and the ITR (HI loop). The PCR products were gel purified and subcloned with the Zero Blunt TOPO kit (Invitrogen). Individual clones were sent for Sanger sequencing (Eton Bioscience) and subcloned into an AAV8 helper plasmid using the appropriate restriction sites. Recombinant AAV8 variants were produced as vectors packaging scCBh-GFP or ssCBA-Luc as described above.

### *In vitro* antibody and serum neutralization assays.

Viral vectors packaging ssCBA-Luc and either monoclonal antibody or serum were diluted in serum-free DMEM, and equal volumes (25 μl each) were combined in black, clear-bottom 96-well plates (Corning) and incubated at room temperature for 30 min. HuH7 cells were added to each well (10,000 cells in 50 μl DMEM, 10% FBS) and incubated at 37°C in 5% CO_2_ for 24 (100,000 vg/cell) or 48 (10,000 vg/cell) h, as indicated for individual experiments. Cells were lysed by incubation in 1× passive lysis buffer (Promega) for 30 min at room temperature. Luciferase activity was measured with a Victor X3 plate reader (PerkinElmer) immediately following the addition of 25 μl of luciferin (Promega).

### *In vivo* characterization of AAV8 mutants.

AAVhum.8 was compared to the parental AAV8 in FRG knockout humanized liver chimeric mice (Yecuris) (37). Vectors packaging scCBh-GFP were administered via retro-orbital injection at a dose of 1 × 10^12^ vector genomes per mouse. Four weeks postinjection, livers were harvested and analyzed to determine vector transduction efficiency in human and mouse hepatocytes. Liver sections were immunostained for GFP and either human or mouse albumin. The number of GFP-positive human and mouse hepatocytes was manually counted for 10 fields of view per animal obtained using a confocal fluorescence microscope and plotted in GraphPad Prism.

### Statistical analysis.

Statistical analysis was performed with the GraphPad Prism software by unpaired Student's *t* test. Statistical significance was determined using the Holm-Sidak method, with alpha = 0.05. *P* values shown in the figures are the following: *, *P* < 0.05; **, *P* < 0.01; ***, *P* < 0.001; and ****, *P* < 0.0001. Error bars represent 1 standard deviation.
